# Comparative Outcomes of Direct Impella Use vs. Intra-Aortic Balloon Pump (IABP)-to- Impella Escalation Strategy in Cardiogenic Shock: Insights from the National Inpatient Sample

**DOI:** 10.21203/rs.3.rs-9887872/v1

**Published:** 2026-07-07

**Authors:** Xiaojia Lu, Aditi Patel, Romani Wahba, Peng Cai, Omar Khalil, Racha Ghousaini, Kirollos Gabrah, Inna Tchoukina, Melissa Smallfield, Michael C Kontos, Keyur B Shah, Pengyang Li

**Affiliations:** The First Affiliated Hospital of Shantou University Medical College; The Pauley Heart Center, Department of Internal Medicine, Virginia Commonwealth University; The Pauley Heart Center, Department of Internal Medicine, Virginia Commonwealth University; Mathematical Sciences, Worcester Polytechnic Institute; Virginia Commonwealth University; Virginia Commonwealth University; The Pauley Heart Center, Department of Internal Medicine, Virginia Commonwealth University; The Pauley Heart Center, Department of Internal Medicine, Virginia Commonwealth University; The Pauley Heart Center, Department of Internal Medicine, Virginia Commonwealth University; The Pauley Heart Center, Department of Internal Medicine, Virginia Commonwealth University; The Pauley Heart Center, Department of Internal Medicine, Virginia Commonwealth University; The Pauley Heart Center, Department of Internal Medicine, Virginia Commonwealth University

**Keywords:** Impella, intra-aortic balloon pump, cardiogenic shock, mortality, costs

## Abstract

**BACKGROUND:**

Impella and IABP are widely used temporary mechanical circulatory support devices in cardiogenic shock (CS). While the Impella is used as an initial support or after IABP as escalation strategy, comparative data on these two strategies remains limited.

**METHODS:**

Using ICD-10 codes in the 2016–2022National Inpatient Sample (NIS) database, we identified patients with primary diagnosis of CS who received Impella support and divided in two groups: direct Impella use and escalation from IABP to Impella. Propensity score matching was performed to adjust for confounding.

**RESULTS:**

Among 107,007 patients were included, 700 (6.5%) underwent IABP-to-Impella escalation strategy. Before matching, the direct Impella use had significantly shorter length-of-stay (LOS) 11.4±13.97 vs. 19.28±20.70 days, *P*<0.001), lower total hospital charges ($441,236 vs. $726,021, *P*<0.001) and fewer transfusions (14.8% vs. 20.6%, *P*<0.001), while the escalation Impella use after IABP had higher rates of durable left ventricular assist device (LVAD) implantation (7.9% vs. 2.2%, *P*<0.001) and rates of orthotopic heart transplant (OHT) (5.0% vs. 1.2%, *P*<0.001). After matching, the direct Impella group still had shorter LOS (13.77±17.21 vs 19.22±20.70 days, *P*<0.001), lower total costs ($478,526 vs. $725,276, *P*<0.001), and fewer transfusions (1.9% vs. 4.9%, *P*=0.026), while escalation Impella use after IABP still had higher rates of LVAD implantation (7.7% vs. 3.0%, *P*<0.001) and OHT (4.9% vs. 1.9%, *P*=0.003).

**CONCLUSIONS:**

In cardiogenic shock, direct and escalation Impella use were associated with similar in-hospital mortality, but direct use was linked to shorter hospital stays and lower costs, highlighting potential benefits in resource utilization and efficiency.

## INTRODUCTION

Cardiogenic shock (CS) is a life-threatening syndrome of inadequate tissue perfusion due to severe cardiac dysfunction, leading to high rates of in-hospital mortality, often between 30% and 50%.[1–4] Although the majority of prior research has focused on acute myocardial infarction (AMI)-associated CS, recent observational studies suggest that non-ischemic etiologies, such as acute decompensated heart failure-CS (HF-CS), account for more than one-half of all CS causes.[5]

Temporary mechanical circulatory support (tMCS) devices are used to stabilize hemodynamics and preserve end-organ perfusion in patients with CS. The intra-aortic balloon pump (IABP) has historically been the most used tMCS device, largely due to its safety profile, ease of insertion, and wide availability.[1] However, its efficacy has been increasingly questioned following the IABP-SHOCK II trial, which demonstrated no mortality benefit from IABP use in patients with AMI-associated CS, leading to a decline in its use.[6, 7] In nonischemic cardiogenic shock, IABP may still provide modest hemodynamic improvements such as increased cardiac index and reduced systemic vascular resistance, though outcomes remain poor. In a single-center study of patients with non-AMI CS, 28% of those treated with IABP either died or required urgent tMCS escalation.[8]

Impella, a percutaneous microaxial flow pump, offers more potent hemodynamic support by actively unloading the left ventricle and increasing forward cardiac output. Its use has expanded rapidly in both AMI- and heart failure (HF)-related cardiogenic shock.[9] The DanGer Shock trial, a multicenter randomized controlled study, showed improved 180-day survival with early use of Impella CP in AMI-CS, although higher complication rates were observed.[10] More recently, multi-center registry studies evaluating Impella 5.0 and 5.5 have shown favorable in-hospital and mid-term survival rates, particularly when used as standalone support in patients with HF-related CS.[11, 12] In clinical practice, the Impella can be used as an initial support device or for stepwise escalation following IABP use in the setting of clinical deterioration.[13, 14] While escalation may reflect institutional preferences or resource limitations, early Impella placement may provide more rapid hemodynamic stabilization.[8–11] Nonetheless, real-world comparative data evaluating these two strategies remain limited.

In this study, we utilized the National Inpatient Sample (NIS) to compare in-hospital outcomes between direct Impella use and IABP-to-Impella escalation strategies among patients hospitalized with cardiogenic shock.

## METHODS

### Data Source and Study Population:

**Figure 1:**
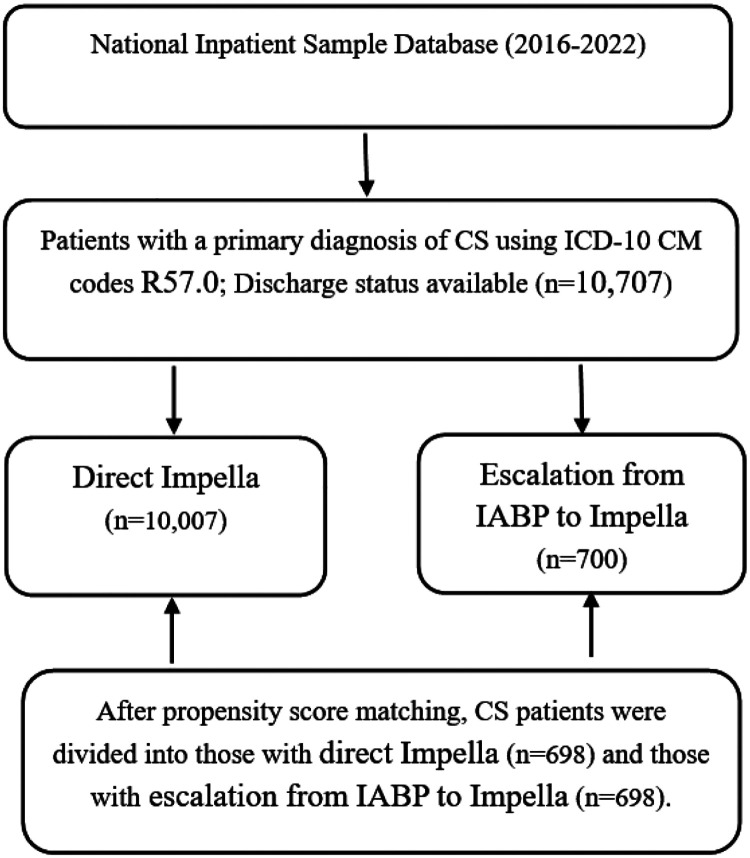
Flow chart of the selection process for the final patient sample used in this study. Inclusion criteria were applied to the National Inpatient Sample 2016–2022 database. All eligible patients were matched 1:1 based on propensity scoring to generate direct Impella use versus escalation from IABP to Impella comparison cohorts. ICD-10-CM codes: Tenth Revision, Clinical Modification Codes.

We conducted a retrospective cohort study using NIS, the largest publicly available all-payer inpatient database in the United States, from 2016–2022. Adult patients (≥ 18 years) hospitalized with cardiogenic shock were identified using ICD-10-CM diagnosis code, R57.0. Patients who received extracorporeal membrane oxygenation (ECMO) during the index hospitalization were excluded to reduce heterogeneity and focus on non-ECMO tMCS strategies. (All ICD-10 code were showed in Supplementary Table 1). The patient selection process in this study is shown in Fig. 1.

### Exposure Definition:

Patients identified as having cardiogenic shock were classified into two groups based on sequence of tMCS device use: (1) Direct Impella Use: patients implanted with the Impella device directly without prior IABP support during the hospitalization (n = 10,007); (2) Escalation Impella Use: patients implanted with Impella after IABP support was initiated during the same hospitalization (n = 700). Impella device types (Impella 2.5, CP 5.0, LD 5.5) were identified using ICD-10-PCS procedure codes and corresponding diagnostic-related group (DRG) classifications, as described in a prior study.[15]

To assess whether device sequencing influenced outcomes across different etiologies of cardiogenic shock, patients were further stratified into two subgroups: AMI-associated CS (n = 7,886) and non-AMI–associated CS (n = 2,821).

### Outcomes:

The primary outcomes were in-hospital mortality, and rates of orthotopic heart transplantation (OHT) and durable left ventricular assist device (LVAD) implantation during the index admission. Secondary outcomes included hospital length of stay (LOS), total hospitalization costs, and rates of blood transfusion.

### Statistical Analysis:

We used mean ± SD to express continuous variables and percentages to express categorical variables for baseline sociodemographic, clinical and hospital-level characteristics. We also provided the median and interquartile range for the LOS and total admission charges. Continuous variables were tested with a *t* test or Wilcoxon rank-sum test as appropriate. Categorical variables were tested with an χ^2^ test or Fisher’s exact test as appropriate. The significance threshold was set at an alpha < 0.05, without adjustment for multiplicity. All data processing and statistical analyses were completed using R (version 4.4.0).

To reduce selection bias, we performed propensity score matching (PSM) in a 1:1 ratio using a nearest-neighbor algorithm with a caliper of 0.2 on the logit of the propensity score. A multivariate logistic regression model was then constructed and adjusted for patient demographics, hospital characteristics, and common comorbidities. Outcomes were compared both before and after propensity matching.

The primary analysis compared outcomes between patients who received any type of Impella device (2.5, CP, or 5.5) as direct support and those who underwent an IABP-to-Impella 5.5 escalation strategy. Given the FDA approval of Impella 5.5 in September 2019 for extended use up to 14 days and its pivotal role in prolonged hemodynamic support, a sensitivity analysis was also conducted, comparing patients treated with IABP followed by Impella 5.5 vs. those who received Impella 5.5 directly, to better reflect patterns of device use in clinical practice. In addition, subgroup analyses were performed by shock etiology (AMI vs. non-AMI) to evaluate whether outcomes differed by underlying cause. AMI-associated cardiogenic shock was defined by a primary diagnosis of acute myocardial infarction using ICD-10-CM codes, as used in a prior study, while all other cases were classified as non-AMI cardiogenic shock.

## RESULTS

### Direct Impella Use VS. IABP-to-Impella Escalation

#### Baseline Characteristics:

The study included 10,707 patients; of those, 700 (7.0%) had an Impella placed after IABP (Escalation Use), leaving 10,007 patients who had an Impella placed directly during their index hospitalization (Direct Use). After propensity score matching, there were 698 patients in both the direct use and escalation use of Impella groups. Baseline characteristics are provided in Table 1.

Prior to matching, age (65.13 vs. 65.45 years, P = 0.52), distribution of sex (28.3% vs. 29.3% female, P = 0.59), and distribution of race/ethnicity (69.2% vs. 65.1% self-reported White, P = 0.145) were similar between groups. Several differences were seen in socioeconomic factors, including patient location, hospital type, hospital bed size, in both groups before matching (Table 1). Escalation use of Impella after IABP was more likely used in patients living in metropolitan counties with large populations, and patients are more likely admitted in urban teaching hospitals and large-sized hospitals. Before matching, patients in the direct use of Impella group had higher rates of hypertension. In contrast, a higher proportion of patients in the escalation use of Impella after initial IABP had chronic heart failure, diabetes mellitus, chronic kidney disease, and acute kidney injury.

After matching, the direct and escalation use of Impella groups were well-matched for all baseline characteristics (*P* > 0.05; Table 1) with balanced groups. All baseline variables in this study had standard mean differences < 0.1 between 2 groups.

### Mortality, LOS, and Total Costs:

In the unmatched cohort (Table 2), there was no difference for the in-hospital mortality rate between IABP-to-Impella escalation use vs. Direct Impella use (39.0% vs. 39.4%, *P* = 0.86), while the direct Impella group had significantly shorter LOS (11.14 ± 13.97 vs. 19.28 ± 20.70 days, *P* < 0.001) and lower total hospital charges ($441,235.84 ± 443,836.22 vs. $726,021.00 ± 743,770.74), *P* < 0.001).

After propensity score matching, these differences remained: the direct Impella group had shorter LOS (13.17 ± 17.21 vs.19.22 ± 20.70 days, *P* < 0.001) and lower total charges ($478,526.21 ± 498,122.44 vs. $725,275.98 ± 744,677.39, *P* < 0.001) with no significant difference in mortality (38.4% vs. 39.1%, *P* = 0.826) (Table 2).

### Bridging to OHT and LVAD Implantation, and Transfusions

Before matching (Table 2), there were higher rates of bridging to LVAD (7.9% vs. 2.2%, *P* < 0.001) and transplant (5.0% vs. 1.2%, *P* < 0.001) and more transfusions (20.6% vs 14.8%, *P* < 0.001) in the escalation group from IABP-to-Impella than those in the direct Impella use. After propensity matching, the higher rates of bridging to LVAD (7.7% vs. 3.0%, *P* < 0.001) and transplant (4.9% vs. 1.9%, *P* < 0.003), and more transfusions (20.5% vs. 15.8%, *P* = 0.026) in the escalation use vs. direct use of Impella remained.

### AMI-associated CS subgroup

Among 7,886 patients with AMI-associated CS, 494 underwent IABP-to-Impella escalation. Baseline characteristics differed between groups but were balanced after matching (Supplementary Table 2). Mortality was similar between groups in both unmatched and matched cohorts (39.3% vs. 40.9%, P = 0.50; matched: 39.4% vs. 37.1%, P = 0.51). Direct Impella use was associated with shorter LOS and lower costs. Before matching, escalation patients had higher LVAD use and transfusion rates; these differences were no longer significant after matching (Supplementary Table 3).

### NAMI-associated CS subgroup

In the NAMI-associated CS subgroup, 2,615 patients received direct Impella support and 206 underwent escalation. Baseline differences were observed but resolved after matching (Supplementary Table 4). Mortality did not differ significantly (38.3% vs. 35.2%, P = 0.40; matched: 38.9% vs. 31.8%, P = 0.17). Direct Impella use, however, was consistently associated with shorter LOS and lower costs. Escalation patients had higher rates of LVAD implantation and transfusions, which remained significant after matching (Supplementary Table 5).

### Direct Impella 5.0/LD/5.5 Use vs. IABP-to-Impella 5.0/LD/5.5 Escalation: Sensitivity Analysis

#### Baseline Characteristics:

We completed a sensitivity analysis comparing baseline characteristics and outcomes in patients who received the surgically-implanted Impella 5.0/LD/5.5 directly vs. after IABP. This included 734 patients; of those, 109 (14.9%) had an Impella 5.0/LD/5.5 placed after IABP (Escalation Use), leaving 625 patients who had an Impella 5.0/LD/5.5 placed directly during their index hospitalization (Direct Use). Differences were also observed in hospital characteristics, but groups were well balanced after propensity matching (all standardized mean differences < 0.1; Table 3).

### Mortality, LOS, and Total Costs:

In the unmatched cohort, mortality did not differ significantly (7.3% vs 13.6%, *P* = 0.097), but direct Impella use was associated with shorter LOS (34.51 ± 26.21 vs. 47.09 ± 29.38 days, *P* < 0.001) and lower total hospital charges ($1,205,100.49 ± 1,048,724.01 vs. $1,655,318.73 ± 1,323,391.83), *P* < 0.001). After propensity score matching, these differences remained: the direct Impella group had shorter LOS (39.01 ± 28.32 vs. 47.16 ± 29.65 days, *P* = 0.041) and lower total charges ($1,331,529.75 ± 1,065,605.45 vs. $1661216.64 ± 1334846.82, *P* = 0.047) with no difference in mortality (Table 4).

### Bridging to OHT and LVAD Implantation, and Transfusions

Before matching, escalation patients had higher rates of LVAD implantation (50.5% vs. 35.8%, P = 0.005) and OHT (32.1% vs. 18.7%, P = 0.002). After matching, these differences were no longer significant. Transfusion rates did not differ between groups in either cohort (Table 4).

## DISCUSSION

To our knowledge, this is the first study to compare escalation Impella use after initial IABP with direct Impella use in patients with cardiogenic shock using a large, nationally representative hospital discharge database. In the unmatched cohort, in-hospital mortality was similar between groups, and this finding persisted after propensity matching. However, direct Impella use was consistently associated with shorter hospital stays and lower total costs. These patterns were consistent in both AMI- and non-AMI–associated subgroups, with no significant mortality difference but persistently shorter LOS and lower costs in the direct Impella group. A sensitivity analysis restricted to surgically implanted Impella 5.0/LD/5.5 confirmed the main findings, showing no difference in mortality but shorter LOS and lower costs with direct Impella use. Together, these results highlight that although escalation from IABP to Impella is a common real-world strategy, it may not yield clinical advantages over early direct Impella support.

Temporary mechanical circulatory support (tMCS) is increasingly used in the management of CS, yet its role remains debated. Guidelines caution against routine use of tMCS in all patients,[16] and most evidence has historically centered on AMI-associated CS. The IABP-SHOCK II trial demonstrated no survival benefit with IABP in AMI-CS, prompting a major shift in recommendations.[17] While IABP can transiently augment coronary perfusion and reduce afterload, its hemodynamic impact is limited, particularly in severe shock.[18, 19] In contrast, the Impella device provides active, continuous unloading of the left ventricle, improves systemic and coronary perfusion, and reduces myocardial oxygen demand. [20–22] Yet despite these physiological benefits, prior registry studies have found persistently high mortality with Impella, regardless of etiology. [23, 24] Recent ACC guidance supports Impella use in selected patients at SCAI stages B–D,[16] underscoring the importance of timing and patient selection, and is consistent with our observation that direct Impella was more common than escalation strategies.

Our findings extend prior literature in several important ways. First, they provide real-world evidence that escalation from IABP to Impella is associated with increased LOS and costs, without a corresponding mortality benefit, compared with direct Impella use. This is biologically plausible: escalation likely reflects initial treatment failure, cumulative device-related costs, and delays in achieving full circulatory support.

Another factor that may have contributed to the higher hospitalization costs in the escalation group is the greater use of downstream advanced therapies, including heart transplantation and durable LVAD implantation. These resource-intensive interventions likely explain part of the cost gap between escalation and direct Impella strategies. However, in our sensitivity analysis restricted to surgically implanted Impella 5.0/LD/5.5 devices, rates of downstream advanced therapies were similar between groups after matching, yet direct Impella use remained associated with shorter LOS and lower costs. This suggests that the observed cost difference is not solely attributable to downstream advanced therapies, but may also reflect inefficiencies inherent to escalation strategies themselves.

At the same time, registry and observational studies have reported that Impella may improve hemodynamic parameters, end-organ perfusion, and short-term survival compared with IABP or no support. However, these benefits must be carefully weighed against the risks of complications, including bleeding, hemolysis, and vascular injury, which can contribute to higher costs and prolonged hospitalization. Furthermore, in PCI populations, Impella has been shown to be cost-effective in European healthcare systems, but whether this translates to CS populations in the U.S. remains uncertain. Our findings emphasize that the timing of Impella initiation—not merely its use—may be critical to maximizing efficiency and value.

Second, our subgroup analyses add granularity by demonstrating consistent patterns across AMI- and non-AMI–associated CS. Regardless of etiology, direct Impella use was associated with shorter LOS and lower costs, supporting a broader relevance of our findings. These results align with prior data showing that Impella can improve hemodynamics across diverse CS phenotypes, though survival remains driven by disease severity and comorbidity burden.

Third, our sensitivity analysis restricted to Impella 5.0/LD/5.5 adds important context. These surgically implanted devices are increasingly used for prolonged support. By demonstrating similar mortality but more favorable efficiency with direct implantation compared to escalation, our analysis strengthens the argument that sequencing strategy itself—rather than solely patient profile—contributes to resource utilization differences.

For clinicians, these findings suggest that when Impella support is indicated, early direct use may be a more efficient strategy than initial IABP with subsequent escalation. Escalation did not appear to improve survival but was consistently associated with greater resource utilization. Early adoption of Impella could streamline management, shorten hospitalization, and reduce costs, though patient selection remains key. Importantly, our results highlight that device sequencing—not only device choice—should be a focus of clinical decision-making and future guideline refinement.

This study should be interpreted in light of its retrospective design. Device strategy was not randomized, and the specific rationale for choosing IABP with escalation vs. direct Impella was not available. Selection likely reflected treating clinicians’ judgment, patient severity, and institutional practice patterns, introducing potential confounding by indication. Although propensity score matching balanced measured covariates, residual confounding from unmeasured variables—such as hemodynamics, vasoactive support, or left ventricular function—cannot be excluded. Reliance on ICD-10 coding introduces the possibility of misclassification. In addition, the dataset only included in-hospital outcomes, precluding evaluation of longer-term survival, rehospitalization, or functional recovery. Cost estimates reflect the U.S. healthcare system and may not be generalizable internationally. Finally, as with any administrative dataset, granular procedural details, device settings, and operator experience were unavailable, all of which could influence outcomes.

### Conclusions

In this large retrospective cohort of hospitalized patients with CS, fewer patients underwent escalation to Impella after initial IABP implantation compared with direct Impella use. However, the escalation strategy was associated with longer LOS and higher healthcare costs. Given that mortality outcomes were similar, our findings suggest that early optimization of device strategy based on CS phenotype should be considered at the time of admission to ensure appropriate resource allocation. Future studies should focus on developing risk stratification tools that integrate both patient characteristics and device selection. In addition, institutional- and provider-level factors influencing tMCS strategies warrant further investigation to inform and refine clinical guidelines for CS management.

## Supplementary Material

Supplementary Files

This is a list of supplementary files associated with this preprint. Click to download.

- 20260601SupplementaryTable1.doc
- 20260601SupplementaryTable2.docx
- 20260601SupplementaryTable3.docx
- 20260601SupplementrayTable4.docx
- 20260601SupplementrayTable5.docx

## Figures and Tables

**Table 1:** Baseline Characteristics of IABP-to-Impella Escalation vs Direct Impella

Variables	Unmatched Cohort			Propensity-Matched Cohort		
	IABP-to-Impella Escalation	Direct Impella	*P* Value	IABP-to-Impella Escalation	Direct Impella	*P* Value
**n**	700	10007		698	698	
**Age, (mean (sd))**	65.45 (12.55)	65.13 (12.76)	0.521	65.48 (12.55)	65.87 (12.76)	0.564
**Sex, n (%)**			0.59			0.953
Female	205 (29.3)	2828 (28.3)		204 (29.2)	206 (29.5)	
**Race, n (%)**			0.145			0.152
White	456 (65.1)	6925 (69.2)		456 (65.3)	474 (67.9)	
Black	80 (11.4)	1010 (10.1)		80 (11.5)	85 (12.2)	
Hispanic	76 (10.9)	818 (8.2)		74 (10.6)	59 (8.5)	
Asian/Pacific Islander	26 (3.7)	336 (3.4)		26 (3.7)	11 (1.6)	
Native American	5 (0.7)	70 (0.7)		5 (0.7)	7 (1.0)	
Other	31 (4.4)	406 (4.1)		31 (4.4)	38 (5.4)	
**Patient location, n (%)**			0.005			0.79
“Central” counties of metro areas of > = 1 million population	205 (29.3)	2621 (26.2)		204 (29.2)	217 (31.1)	
“Tinge” counties of metro areas of > = 1 million population	198 (28.3)	2383 (23.8)		197 (28.2)	178 (25.5)	
Counties in metro areas of 250,000–999,999 population	126 (18.0)	2364 (23.6)		126 (18.1)	141 (20.2)	
Counties in metro areas of 50,000–249,999 population	60 (8.6)	940 (9.4)		60 (8.6)	51 (7.3)	
Micropolitan counties	57 (8.1)	921 (9.2)		57 (8.2)	57 (8.2)	
Non metropolitan or micropolitan counties	52 (7.4)	734 (7.3)		52 (7.4)	51 (7.3)	
NA	2 (0.3)	44 (0.4)		2 (0.3)	3 (0.4)	
**Mean household income, n (%)**			0.595			0.989
$1–$42,999	196 (28.0)	2950 (29.5)		195 (27.9)	191 (27.4)	
$43,000–$53,999	176 (25.1)	2605 (26.0)		176 (25.2)	185 (26.5)	
$54,000–$70,999	164 (23.4)	2352 (23.5)		163 (23.4)	160 (22.9)	
$71,000 or more	150 (21.4)	1897 (19.0)		150 (21.5)	148 (21.2)	
Unknown	14 (2.0)	203 (2.0)		14 (2.0)	14 (2.0)	
**Primary payer, n (%)**			0.073			0.679
Medicare	401 (57.3)	5466 (54.6)		400 (57.3)	403 (57.7)	
Medicaid	69 (9.9)	1096 (11.0)		69 (9.9)	57 (8.2)	
Private including HMO	192 (27.4)	2577 (25.8)		191 (27.4)	187 (26.8)	
Self-pay	20 (2.9)	506 (5.1)		20 (2.9)	27 (3.9)	
No charge	1 (0.1)	30 (0.3)		1 (0.1)	1 (0.1)	
Other	17 (2.4)	315 (3.1)		17 (2.4)	23 (3.3)	
**Hospital type, n (%)**			< 0.001			0.862
Rural	8 (1.1)	308 (3.1)		8 (1.1)	10 (1.4)	
Urban non-teaching	62 (8.9)	1547 (15.5)		62 (8.9)	59 (8.5)	
Urban teaching	630 (90.0)	8152 (81.5)		628 (90.0)	629 (90.1)	
**Hospital Region, n (%)**			0.159			0.588
Northeast	112 (16.0)	1452 (14.5)		112 (16.0)	99 (14.2)	
Midwest	143 (20.4)	1829 (18.3)		142 (20.3)	153 (21.9)	
South	301 (43.0)	4370 (43.7)		300 (43.0)	313 (44.8)	
West	144 (20.6)	2356 (23.5)		144 (20.6)	133 (19.1)	
**Hospital Bed Size, n (%)**			0.007			0.385
Small	59 (8.4)	1190 (11.9)		59 (8.5)	74 (10.6)	
Medium	174 (24.9)	2640 (26.4)		174 (24.9)	173 (24.8)	
Large	467 (66.7)	6177 (61.7)		465 (66.6)	451 (64.6)	
**Comorbidities, n (%)**						
Smoking	234 (33.4)	3593 (35.9)	0.2	234 (33.5)	237 (34.0)	0.91
Hypertension	69 (9.9)	1535 (15.3)	< 0.001	69 (9.9)	76 (10.9)	0.599
DM	331 (47.3)	4245 (42.4)	0.013	329 (47.1)	322 (46.1)	0.748
Hyperlipidemia	355 (50.7)	5022 (50.2)	0.817	354 (50.7)	347 (49.7)	0.748
Obesity	151 (21.6)	1987 (19.9)	0.294	150 (21.5)	146 (20.9)	0.844
CAD	515 (73.6)	7225 (72.2)	0.459	514 (73.6)	499 (71.5)	0.401
CHF	632 (90.3)	7463 (74.6)	< 0.001	630 (90.3)	612 (87.7)	0.146
AF	254 (36.3)	3280 (32.8)	0.062	252 (36.1)	260 (37.2)	0.697
OSA	75 (10.7)	860 (8.6)	0.064	74 (10.6)	70 (10.0)	0.792
CKD	280 (40.0)	3037 (30.3)	< 0.001	278 (39.8)	282 (40.4)	0.87
COPD	118 (16.9)	1543 (15.4)	0.336	118 (16.9)	112 (16.0)	0.718
Vent arrhythmia n (%)	298 (42.6)	4310 (43.1)	0.827	297 (42.6)	301 (43.1)	0.871
AKI, n (%)	515 (73.6)	6127 (61.2)	< 0.001	513 (73.5)	515 (73.8)	0.952
ARF, n (%)	434 (62.0)	6487 (64.8)	0.141	434 (62.2)	456 (65.3)	0.242
ALI n (%)	180 (25.7)	2305 (23.0)	0.115	180 (25.8)	194 (27.8)	0.432

AF, Atrial Fibrillation; AKI, Acute Kidney Injury; ALI, Acute Liver Injury; ARF, Acute Respiratory Failure; CAD, Coronary Artery Disease; CHF, Congestive Heart Disease; CKD, Chronic Kidney Disease; COPD, Chronic Obstructive Pulmonary Disease; COVID-19: Coronavirus Disease 2019; DM, Diabetes Mellitus; OSA, Obstructive Sleep Apnea; Vent arrhythmia, Ventricular Arrthymia.

**Table 2:** In-Hospital Outcomes of IABP-to-Impella Escalation vs Direct Impella

	Unmatched Cohort		Propensity-Matched Cohort			
Variables	IABP-to-Impella Escalation	Direct Impella	*P* Value	IABP-to-Impella Escalation	Direct Impella	*P* Value
**n**	700	10007		698	698	
**Primary Outcomes**						
Death, n (%)	273 (39.0)	3944 (39.4)	0.860	273 (39.1)	268 (38.4)	0.826
LVAD n (%)	55 (7.9)	224 (2.2)	< 0.001	54 (7.7)	21 (3.0)	< 0.001
OHT n(%)	35 (5.0)	117 (1.2)	< 0.001	34 (4.9)	13 (1.9)	0.003
**Secondary Outcomes**						
LOS, (mean (sd))	19.28 (20.70)	11.14 (13.97)	< 0.001	19.22 (20.70)	13.17 (17.21)	< 0.001
Total charge (mean (sd))	726021.00(743770.74)	441235.84(443836.22)	< 0.001	725275.98(744677.39)	478526.21(498122.44)	< 0.001
Transfusions n (%)	144 (20.6)	1479 (14.8)	< 0.001	143 (20.5)	110 (15.8)	0.026

LOS, Length of Stay; LVAD, Left Ventricular Assist Device; OHT, Heart Transplant.

**Table 3:** Baseline Characteristics of IABP-to- Impella 5.0/LD/5.5 Escalation vs Direct Impella 5.5/LD/5.5

Variables	Unmatched Cohort			Propensity-Matched Cohort		
	IABP-to-Impella 5.0/LD/5.5 Escalation	Direct Impella 5.0/LD/5.5	*P* Value	IABP-to-Impella 5.0/LD/5.5 Escalation	Direct Impella 5.0/LD/5.5	*P* Value
**n**	109	625		107	107	
**Age, (mean (sd))**	56.61 (13.81)	59.07 (13.36)	0.078	56.36 (13.81)	57.61 (12.87)	0.497
**Sex, n (%)**			0.569			0.201
Female	22 (20.2)	145 (23.2)		22 (20.6)	14 (13.1)	
**Race, n (%)**			.055			0.918
White	56 (51.4)	408 (65.3)		56 (52.3)	55 (51.4)	
Black	23 (21.1)	94 (15.0)		23 (21.5)	20 (18.7)	
Hispanic	16 (14.7)	45 (7.2)		15 (14.0)	15 (14.0)	
Asian/Pacific Islander	5 (4.6)	21 (3.4)		4 (3.7)	6 (5.6)	
Native American	0 (0.0)	1 (0.2)		4 (3.7)	3 (2.8)	
Other	4 (3.7)	18 (2.9)		5 (4.7)	8 (7.5)	
**Patient location, n (%)**			.28			0.889
“Central” counties of metro areas of > = 1 million population	40 (36.7)	161 (25.8)		38 (35.5)	31 (29.0)	
“Finge” counties of metro areas of > = 1 million population	29 (26.6)	178 (28.5)		29 (27.1)	28 (26.2)	
Counties in metro areas of 250,000–999,999 population	16 (14.7)	131 (21.0)		16 (15.0)	20 (18.7)	
Counties in metro areas of 50,000–249,999 population	8 (7.3)	65 (10.4)		8 (7.5)	11 (10.3)	
Micropolitan counties	9 (8.3)	51 (8.2)		9 (8.4)	10 (9.3)	
Non metropolitan or micropolitan counties	7 (6.4)	36 (5.8)		7 (6.5)	7 (6.5)	
NA	0 (0.0)	3 (0.5)		38 (35.5)	31 (29.0)	
**Mean household income, n (%)**			0.425			0.813
$1–$42,999	31 (28.4)	168 (26.9)		31 (29.0)	32 (29.9)	
$43,000–$53,999	22 (20.2)	155 (24.8)		22 (20.6)	18 (16.8)	
$54,000–$70,999	33 (30.3)	156 (25.0)		32 (29.9)	39 (36.4)	
$71,000 or more	20 (18.3)	138 (22.1)		19 (17.8)	15 (14.0)	
Unknown	3 (2.8)	8 (1.3)		3 (2.8)	3 (2.8)	
**Primary payer, n (%)**			0.861			0.706
Medicare	45 (41.3)	270 (43.2)		43 (40.2)	50 (46.7)	
Medicaid	11 (10.1)	79 (12.6)		11 (10.3)	12 (11.2)	
Private including HMO	47 (43.1)	233 (37.3)		47 (43.9)	37 (34.6)	
Self-pay	2 (1.8)	20 (3.2)		2 (1.9)	2 (1.9)	
No charge	0 (0.0)	2 (0.3)				
Other	4 (3.7)	20 (3.2)		4 (3.7)	6 (5.6)	
**Hospital type, n (%)**			.01			N/A
Rural	0 (0.0)	10 (1.6)				
Urban non-teaching	0 (0.0)	39 (6.2)				
Urban teaching	109 (100.0)	576 (92.2)				
**Hospital Region, n (%)**			0.045			0.868
Northeast	18 (16.5)	122 (19.5)		18 (16.8)	17 (15.9)	
Midwest	24 (22.0)	90 (14.4)		23 (21.5)	19 (17.8)	
South	52 (47.7)	269 (43.0)		51 (47.7)	53 (49.5)	
West	15 (13.8)	144 (23.0)		15 (14.0)	18 (16.8)	
**Hospital Bed Size, n (%)**			0.049			0.621
Small	3 (2.8)	44 (7.0)		3 (2.8)	5 (4.7)	
Medium	13 (11.9)	113 (18.1)		13 (12.1)	16 (15.0)	
Large	93 (85.3)	468 (74.9)		91 (85.0)	86 (80.4)	
**Comorbidities, n (%)**						
Smoking	24 (22.0)	148 (23.7)	0.798	23 (21.5)	28 (26.2)	0.521
Hypertension	4 (3.7)	40 (6.4)	0.374	4 (3.7)	6 (5.6)	0.746
DM	47 (43.1)	235 (37.6)	0.324	46 (43.0)	52 (48.6)	0.493
Hyperlipidemia	36 (33.0)	240 (38.4)	0.336	35 (32.7)	38 (35.5)	0.773
Obesity	16 (14.7)	124 (19.8)	0.257	16 (15.0)	17 (15.9)	1
CAD	36 (33.0)	311 (49.8)	0.002	36 (33.6)	38 (35.5)	0.886
CHF	107 (98.2)	568 (90.9)	0.017	105 (98.1)	105 (98.1)	1
AF	44 (40.4)	282 (45.1)	0.414	44 (41.1)	50 (46.7)	0.491
OSA	15 (13.8)	69 (11.0)	0.509	14 (13.1)	16 (15.0)	0.844
CKD	47 (43.1)	224 (35.8)	0.178	45 (42.1)	41 (38.3)	0.676
COPD	13 (11.9)	73 (11.7)	1	13 (12.1)	17 (15.9)	0.555
Vent arrhythmia n (%)	57 (52.3)	333 (53.3)	0.931	56 (52.3)	60 (56.1)	0.681
AKI, n (%)	95 (87.2)	474 (75.8)	0.013	93 (86.9)	93 (86.9)	1
ARF, n (%)	52 (47.7)	372 (59.5)	0.028	50 (46.7)	46 (43.0)	0.68
ALI n (%)	34 (31.2)	156 (25.0)	0.21	33 (30.8)	23 (21.5)	0.162

AF, Atrial Fibrillation; AKI, Acute Kidney Injury; ALI, Acute Liver Injury; ARF, Acute Respiratory Failure; CAD, Coronary Artery Disease; CHF, Congestive Heart Disease; CKD, Chronic Kidney Disease; COPD, Chronic Obstructive Pulmonary Disease; COVID-19: Coronavirus Disease 2019; DM, Diabetes Mellitus; OSA, Obstructive Sleep Apnea; Vent arrhythmia, Ventricular Arrthymia.

**Table 4:** In-Hospital Outcomes of IABP-to- Impella 5.0/LD/5.5 Escalation vs Direct Impella 5.5/LD/5.5

Variables	Unmatched Cohort		Propensity-Matched Cohort			
	IABP-to- Impella 5.0/LD/5.5 Escalation	Direct Impella 5.0/LD/5.5	*P* Value	IABP-to- Impella 5.0/LD/5.5 Escalation	Direct Impella 5.0/LD/5.5	*P* Value
**n**	109	625		107	107	
**Primary Outcomes**						
Death, n (%)	8 (7.3)	85 (13.6)	0.097	8 (7.5)	8 (7.5)	1
LVAD n (%)	55 (50.5)	224 (35.8)	0.005	53 (49.5)	49 (45.8)	0.681
OHTn(%)	35 (32.1)	117 (18.7)	0.002	35 (32.7)	30 (28.0)	0.552
**Secondary Outcomes**						
LOS, (mean (sd))	47.09 (29.38)	34.51 (26.21)	< 0.001	47.16 (29.65)	39.01 (28.32)	0.041
Total charge (mean (sd))	1655318.73(1323391.83)	1205100.49(1048724.01)	< 0.001	1661216.64(1334846.82)	1331529.75(1065605.45)	0.047
Transfusions n (%)	21 (19.3)	149 (23.8)	0.357	21 (19.6)	23 (21.5)	0.866

LOS, Length of Stay; LVAD, Left Ventricular Assist Device; OHT, Heart Transplant.

## Data Availability

The data that support the findings of this study are available from NIS database but restrictions apply to the availability of these data, which were used under license for the current study, and so are not publicly available.

## ABBREVIATIONS

CS: cardiogenic shock
IABP: intra-aortic balloon pump
LOS: length of stay
LVAD: left ventricular assist device
NIS: National Inpatient Sample
OHT: orthotopic heart transplant
tMCS: temporary mechanical circulatory support
UNOS: United Network for Organ Sharing
